# Reviewed article: Silva GO, Braga YS, Reis IF, Alessio Junior LE, Raia VA, Prevedello AS, Alessio AM. Mortalidade por Câncer de Colo do Útero no Mato Grosso, 2013-2022: estudo descritivo e análise de série temporal. Epidemiol Serv Saude. 2025:34;e20240503.

**DOI:** 10.1590/S2237-96222025v34e20240503.a

**Published:** 2025-08-08

**Authors:** Samuel Lopes dos Santos

**Table:** 

Rating	Excellent	Good	Average	Below average	Poor
Interest		✓			
Quality			✓		
Originality		✓			
Overall			✓		

**Table:** 

Questionnaire	Yes	No	Not applicable
Does the manuscript contain new and significant information to justify publication?	✓		
Does the Abstract (Summary) clearly and accurately describe the content of the article?	✓		
Is the problem significant and concisely stated?	✓		
Are the methods described comprehensively?	✓		
Are the interpretations and conclusions justified by the results?	✓		
Is adequate reference made to other work in the field?	✓		

## Manuscript Structure

Length of article is: Adequate

Number of tables is: Adequate

Number of figures is: Adequate

Please state any conflict(s) of interest that you have in relation to the review of this paper (state “none” if this is not applicable): None

## Comments

Prezado Autor,

Parabéns por trabalhar com essa temática, a análise da mortalidade do câncer do colo do útero a nível regional é inovadora. Alguns pontos do texto geraram dúvidas, abaixo descrevo alguns itens que deverão ser revistos e esclarecidos:

1 – Revise o resumo para que os resultados apresentados sejam apoiados por dados numéricos e tenham seus dados apresentados no tópico métodos, por exemplo a análise descritiva da taxa de mortalidade bruta que não está relatada no resumo.

2- Retorne à introdução, é preciso garantir que fique claro a importância e os resultados esperados ao se analisar o perfil epidemiológico e a tendência da mortalidade dos casos de câncer do colo de útero no estado do Mato Grosso.

3 – No resumo e na introdução foram descritos objetivos de pesquisa diferentes, é necessário definir um único objetivo.

4 – O delineamento do estudo está adequado, entretanto, é necessário o esclarecimento de qual é a fonte dos dados, apesar de ter sido indicado o programa Warehouse, nenhum link de acesso foi citado, não ficando claro se os dados são públicos e de acesso a qualquer pessoa.

5 – Nos métodos não é descrito a padronização da taxa de mortalidade e uma das etapas da pesquisa comparou a taxa de mortalidade entre as macrorregiões de saúde do estado do Mato Grosso, porém a comparação fica inviável já que pode haver o viés de diferenças de idade entre as regiões. É essencial que as taxas sejam padronizadas por idade.

6- Ainda nos métodos, é essencial a caracterização da população e do local da pesquisa, desta forma, é preciso constar no texto, quantas regiões de saúde existem no estado, número de mulheres residentes em cada região, quantos municípios, características de acesso a saúde.

7 – O item discussão deve ser revisitado, sendo necessário esclarecer no texto quais as hipóteses para que os casos de câncer de colo de útero no Mato Grosso sejam maiores em algumas faixas etárias e em algumas macrorregiões de saúde. 

8 – Ainda na discussão, a tendência da mortalidade do câncer de colo de útero está estagnada no estado, mas as hipóteses para este achado não ficam claros, justifique no texto quais as hipóteses para este achado.

Atenciosamente. 

